# Near-infrared spectroscopy, pupillometry and end-tidal CO_2_ as predictive factors of survival at hospital admission after out-of-hospital cardiac arrest

**DOI:** 10.1016/j.resplu.2026.101316

**Published:** 2026-04-09

**Authors:** N. Segond, D. Savary, M. Veloso, P. Bouzat, J. Turk, J.L. Bosson, D. Viglino, J.F. Payen, G. Debaty

**Affiliations:** a Emergency Department and Mobile Intensive Care Unit, University Hospital of Grenoble Alpes and University of Grenoble Alpes, France; CNRS, UMR 5525, VetAgro Sup, Grenoble INP, TIMC, Grenoble, France; b Emergency Department, Angers University Hospital, Angers, Francia. Univ. Angers, Inserm, EHESP, Irset (Institut de recherche en santé, environnement et travail) – UMR_S1085, SFR ICAT, CAPTV-CDC, F-49000 Angers, France; c Univ. Grenoble Alpes, Data Engineering Unit, Public Health Department, Grenoble Alpes University Hospital, Grenoble, France; d Université Grenoble Alpes, Inserm U1216, Grenoble Institut Neurosciences, Department of Anaesthesia and Intensive Care, Centre Hospitalier Universitaire Grenoble, Grenoble, France; e Chambery Metropole Savoie Hospital, Emergency Department and Mobile Intensive Care Unit, Chambery, France; f HP2 Laboratory, INSERM U1300, Univ. Grenoble Alpes, Grenoble, France

**Keywords:** Cardiac arrest, Prognostication, End-tidal CO_2_, Pupillometry, Near-infrared spectroscopy

## Abstract

**Background:**

Early prognostication of out-hospital cardiac arrest (OHCA) patient’s during cardiopulmonary resuscitation (CPR) is challenging and based on a combination of factors. The aim of this study was to assess the prognostic value of cerebral oximetry (rSO_2_), infrared automated pupillometry and end-tidal CO_2_ (EtCO_2_) alone or in combination for patients with OHCA.

**Methods:**

A prospective multicenter cohort study in 3 French prehospital mobile intensive care units was performed. Adult patients with a presumed medical OHCA were included. Measurements were made at medical team arrival every 5 min for at least 15 min. The primary endpoint was the prognostic value of the parameters for survival at hospital admission.

**Results:**

Of the 112 patients included and analyzed, 19 (17%) survived to hospital admission. Initial values for survival at hospital admission differed for rSO_2_ (alive: median: 30.0% (Q1: 19.0; Q3: 43.0) vs dead: 15.0% (15.0; 22.0), *p* < 0.001) and for EtCO_2_ (43.5 mmHg (26.8; 56) vs. 22.0 mmHg (15.0; 36.5), *p* = 0.006) in contrast to pupillometry values (6.0% (2.0; 11.5) vs. 3.0% (0; 7.5), *p* = 0.10). Initial rSO_2_ alone had an AUC of 0.74 (95% CI 0.59–0.88) and EtCO_2_ alone provide an AUC of 0.77 (95% CI 0.64–0.89). Pupillometry alone had an AUC of 0.65 (95% CI 0.51–0.79). In combination, initial EtCO_2_ plus initial rSO_2_ provided an AUC of 0.85 (95% CI 0.73–0.96).

**Conclusion:**

During OHCA, rSO_2_ and EtCO_2_ could be predictive factors for survival at hospital admission with an acceptable discrimination, and their prognostic value was improved when both factors were combined. Further studies are needed to clarify the prognostic value of rSO_2_, EtCO_2_ and pupillometry for survival at hospital admission*.*

## Introduction

Despite efforts to increase survival following out-of-hospital cardiac arrest (OHCA), only 25% of patients are transported with return of spontaneous circulation (ROSC), and only 8% survive to hospital discharge.^1^, ^2^ Early recognition and defibrillation, combined with high-quality chest compressions, are well-known prognostic factors for survival. Moreover, end-tidal CO_2_ (EtCO_2_) during cardiopulmonary resuscitation (CPR) is recognized as an important negative prognostic factor if it is below 10 mmHg after 20 min of CPR.^3^, ^4^ Studies have shown that an increase in EtCO_2_ is associated with ROSC and survival.^5^ However, EtCO_2_ alone appears to be insufficient for prognostication of survival in prehospital conditions.^6^

In recent years, near-infrared spectroscopy, which measures regional cerebral oximetry (rSO_2_), has emerged as a potential prognostic factor for survival in patients with out-of-hospital cardiac arrest^7^ and could be used in the early phase by paramedics.^8^ rSO_2_ may have a similar prognostic value that EtCO_2_, with greater specificity for rSO_2_ and greater sensitivity for EtCO_2_ in predicting ROSC.^6^ However, there is a lack of evidence regarding the optimal threshold value of rSO_2_ for predicting ROSC and survival.

Pupillary light reflex is a common prognostic factor for OHCA patients after ROSC.^9^, ^10^ Pupillometry, which measures pupil diameter using infrared technology, has shown potential predictive value for survival and neurological function in the early phase after ROSC.^11^, ^12^, ^13^ However, infrared pupillometry has never been evaluated for predicting survival before ROSC in OHCA.

As demonstrated in post resuscitation care studies,^14^ multimodal prediction represents an opportunity to better predict outcomes in the early phase of OHCA, aiding in decisions to continue or discontinue CPR.

The aim of the present study was to evaluate the prognostic value of rSO_2_, pupillometry, and EtCO_2_, individually or in combination with other prognostic factors, in predicting survival upon hospital admission for patients with OHCA.

## Methods

### Study design

We conducted a prospective cohort analysis of three French prehospital intensive care units in the French Alps (Grenoble, Chambéry, Annecy) from February 2016 to September 2018. Adult patients with a presumed medical OHCA were included. The exclusion criteria were as follows: age under 18 years, pregnancy, traumatic cardiac arrest, non-eligibility for resuscitation, and patients with restrictive legal rights.

The study was approved by the institutional review board “Sud-Est IV” (Reference: 15-CHUG-32) and registered at *clinicaltrials.gov* (ID: NCT02684955).

### Study system

In France, when a cardiac arrest occurs, both a basic life support (BLS) team and an advanced life support (ALS) team are dispatched. The first responders, trained firefighters, perform BLS, which includes chest compressions, defibrillation, and ventilation. The emergency medical services (EMS), composed of an emergency physician, an emergency nurse, and an ambulance driver, then provide ALS. If ROSC occurs, the patient was transported to the hospital for post-ROSC care. The French Emergency System teams followed the most recent European Resuscitation Guidelines for BLS and ALS. During the study period (2016–2019) in the Northern French Alps Emergency Network (covering Grenoble, Annecy, and Chambéry), the median time from cardiac arrest to firefighter arrival was 8.0 min (IQR: 12.0–17.0), and the median time to EMS team arrival was 15.0 min (IQR: 22.0–33.0). Among the overall cardiac arrest population, the rate of return of spontaneous circulation (ROSC) was 26%; survival to hospital admission was 17%, and the 30-day survival rate was 7.3%.

### Data collection

**Fig. 1:**
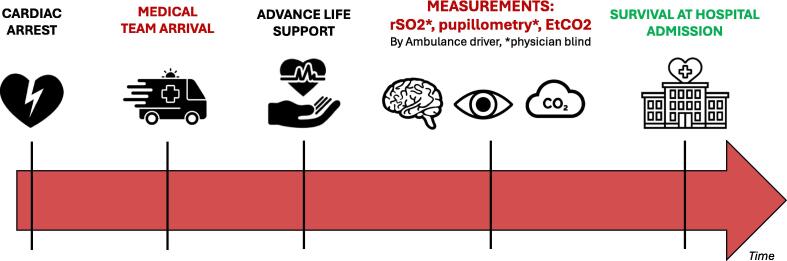
**Description of study protocol**. *rSO_2_: near infrared spectroscopy, EtCO_2_: end-tidal CO_2_*.

For this study, measurements were taken by the ambulance driver to ensure the physician remained blinded to the rSO_2_ and pupillometry values. rSO_2_ was measured using the INVOS 5100 regional oximeter (COVIDIEN®, France), while pupillometry was measured using an infrared automatic pupillometer (IDMED®, France). A dedicated rescue backpack containing both devices was carried by the ambulance driver. Measurements were taken upon arrival (T0), at five minutes (T5), at ten minutes (T10), and at fifteen minutes (T15). The physician and nurse conducted ALS following current guidelines. After tracheal intubation, EtCO_2_ was measured using a multiparameter monitor available to each team, and the physician and nurse were not blinded to EtCO_2_ as it was a known prognostic factor. If ROSC was not achieved, the patient was declared deceased at the scene by the physician. If ROSC occurred, the patient was transported to the hospital and admitted to the intensive care unit for post-ROSC care following current guidelines. All parameters included in the analysis were collected following the Utstein guidelines. Additionally, time intervals between cardiac arrest, the first call to dispatch center, firefighters' on-scene arrival, first shock, and EMS on-scene arrival were recorded. The study design is illustrated in Fig. 1.

### Outcome, objectives and endpoints

The primary outcome was survival to hospital admission, defined as the presence of a spontaneous and sustained pulse at the time of hospital admission. The primary objective was to evaluate the prognostic value of combined rSO_2_ and pupillometry values adjusted on bystander CPR and initial cardiac rhythm for survival to hospital admission. The primary endpoint was the prognostic values (sensitivity, specificity, positive and negative predictive values (PPV and NPV), and the area under the Receiver Operating Characteristic (ROC) curve (AUC) with their 95% confidence intervals) of initial rSO_2_ combined with pupillometry, adjusted on bystander CPR and initial cardiac rhythm for survival to hospital admission (defined as the presence of a spontaneous and sustained pulse upon hospital admission). Secondary analyses included the prognostic value of initial, median, and maximum values for rSO_2_, pupillometry, and EtCO_2_ for survival to hospital admission. To optimized predictive accuracy, these parameters were analyzed both individually and in combination. A temporal analysis was conducted to measure how changes in these values during CPR served as predictors of the survival to hospital admission.

### Number of subjects needed

Initially, the primary endpoint was based on survival with good neurological outcome, defined as a Cerebral Performance Category (CPC) of 1 or 2 at 30 days. It was hypothesized that 1% of patients with rSO_2_ ≤ 30% and absent pupillary reflex would achieve CPC 1 or 2, compared to 15% in patients with rSO_2_ > 30% and/or the presence of pupillary reflex. With an alpha risk of 5% and a power of 80%, the required sample size was calculated to be 135 patients.

Due to recruitment challenges despite a one-year extension and poor neurological outcomes in the population, the primary endpoint was modified to survival at hospital admission. This modification was registered at *clinicaltrials.gov*.

### Data analysis

Quantitative variables were described as mean and standard deviation when normality conditions were met, or as median and interquartile range for skewed distributions. Qualitative variables were presented as numbers and percentages. Missing data were reported but excluded from calculations.

For the primary endpoint, ROC curves were generated for each prognostic factor in univariate analysis to determine optimal thresholds, maximizing the Youden index for predicting survival at hospital admission. A multivariate logistic regression incorporating all prognostic factors was performed, and the model's discrimination ability was assessed using a ROC curve. A Chi-square test was applied to assess the association between survival and the prognostic thresholds identified. If Cochran's criteria were not met, Fisher's exact test was used. Depending on the results, a relative risk with a 95% confidence interval was calculated. Diagnostic test performance indicators, including PPV, NPV, sensitivity, and specificity, were reported.

For secondary analyses, the same statistical approach as the primary outcome was applied. Thresholds for each prognostic factor were visually determined for secondary outcomes, and subsequent analyses followed the same methodology. A logistic regression model was used to evaluate trends (increase, decrease, or stability) in EtCO_2_ and rSO_2_ up to 30 min, in relation to survival at hospital admission.

A time-dependent descriptive analysis was conducted post-hoc, presenting medians and interquartile range for measurements at T0, T5, T10, and T15.

The statistical significance threshold of 5% was used.

This report was prepared in accordance with the STROBE Statement guidelines.^15^

## Results

### Baseline characteristics

Among the three prehospital teams (Grenoble, Chambéry, Annecy), a total of 112 patients were included and analyzed. The median age was 70 years (Q1: 61; Q3: 79). Thirty-eight (34%) patients were women. Bystander-initiated cardiopulmonary resuscitation (CPR) was performed in 72 (66%) cases. A shockable rhythm was observed in 11 (10%) patients, while 77 (70%) presented with asystole. Return of spontaneous circulation (ROSC) was achieved in 32 (29%) patients, and 19 (17%) survived to hospital admission (Table 1).

**Table 1:** Population characteristics.

	**All cases** **(*n* = 112)**	**No survival at hospital admission** **(*n* = 93)**	**Survival at hospital admission** **(*n* = 19)**
Age (years); median [Q1; Q3]	70 [61; 79]	72 [62; 80]	68 [56; 77]
Women; *n* (%)	38 (33.9)	30 (32.3)	8 (42.1)

**Cardiac arrest location: *n* (%)**			
Home	97 (86.6)	83 (89.2)	14 (73.7)
Public place	13 (11.6)	8 (8.6)	5 (26.3)
Other	1 (0.9)	1 (1.1)	0 (0)
Witness cardiac arrest; *n* (%)	76 (67.9)	60 (64.5)	16 (84.2)

**Inclusion center; *n* (%)**			
Grenoble	38 (33.9)	27 (29.0)	11 (57.8)
Chambery	14 (12.5)	12 (12.9)	2 (10.5)
Annecy	60 (53.6)	54 (58.1)	6 (31.6)

**Type of witness: *n* (%)**			
Family	80 (71.4)	67 (72.0)	13 (68.4)
People	14 (12.5)	10 (10.8)	4 (21.1)
Rescuer	9 (8.0)	8 (8.6)	1 (5.3)
EMS	1 (0.9)	1 (1.1)	0 (0)
Other	5 (4.5)	4 (4.3)	1 (5.3)
CPR initiated by witness; *n* (%)	72 (64.3)	56 (60.2)	16 (84.2)

**Etiology of cardiac arrest: *n* (%)**			
Cardiac	75 (67.0)	63 (67.7)	12 (63.2)
Respiratory	16 (14.2)	12 (12.9)	4 (21.1)
Toxic	2 (1.8)	2 (2.2)	0 (0)
Other	5 (4.5)	3 (3.2)	2 (10.5)

**Initial cardiac rhythm; *n* (%)**			
Asystole	77 (68.8)	67 (72.0)	10 (10.8)
Pulseless Electrical Activity	11 (9.8)	9 (9.7)	2 (2.2)
Shockable rhythm (VF, VT)	22 (19.7)	15 (16.1)	7 (7.5)
Signs of life (gasps); *n* (%)	13 (11.6)	9 (9.6)	4 (21.1)

**Pupillary status at EMS arrival; *n* (%)**			
Symmetric pupil	98 (87.5)	80 (86.0)	18 (94.7)
Areactive pupil	95 (84.8)	81 (87.1)	14 (73.7)
Adrenaline dose (mg); median [Q1; Q3]	4 [3; 5]	4 [3; 5]	2 [2; 5]
Amiodarone dose (mg); median [Q1; Q3]	0 [0; 0]	0 [0; 0]	0 [0; 300]

**Delay from cardiac arrest to: (min; median [IQR])**			
Firefighters’ arrival	12 [6;18]	13 [9; 25]	10 [10; 15]
First Shock	18 [12; 24]	18 [12; 30]	13 [7; 15]
EMS arrival	24 [12; 36]	23 [15; 34]	20 [12; 29]
First measurement of EtCO_2_, rSO_2_ and pupillometry	28 [22; 39]	30 [22; 40]	24 [22; 34]

**Delay from first call to: (min; median [IQR])**			
Firefighters’ arrival	12 [6; 12]	9 [7; 13]	10 [7; 13]
First shock	12 [12; 18]	17 [10; 21]	13 [8; 14]
EMS arrival	18 [12; 24]	18 [18; 27]	17 [10; 24]

**Survival outcomes: *n* (%)**			
ROSC	32 (28.6)	13 (13.9)	19 (100.0)
Survival at hospital admission	19 (17.0)	0 (0.0)	19 (100.0)
Survival at hospital discharge	2 (1.8)	0 (0.0)	2 (10.5)
Survival at 30 days	2 (1.8)	0 (0.0)	2 (10.5)
CPC 1 or 2 at 30 days	1 (0.9)	0 (0.0)	1 (5.2)

*EMS: Emergency Medical Services; VF: Ventricular Fibrillation; VT: Ventricular Tachycardia; ROSC: Return to Spontaneous Circulation; CPC: Cerebral Performance Category status.*

*Missing data regarding this table are presented in*Appendix 2*.*

Median initial rSO_2_ was 15.0% (Q1: 15.0; Q3: 27.50) and differed significantly according to survival at hospital admission (alive: 30.0 (19.0; 43.0) vs dead: 15.0 (15.0; 22.0), *p* < 0.001). Median initial EtCO_2_ was 25.0 mmHg (Q1: 16.0; Q3: 39.0) and differed significantly according to survival at hospital admission (alive: 43.5 (26.8; 56) vs. dead: 22.0 (15.0; 36.5), *p* = 0.006). Median initial pupillometry measurement was 3% (Q1: 1.0; Q3: 9.0) and did not differ significantly according to survival at hospital admission (alive: 6.0 (2.0; 11.5) vs. dead: 3.0 (0; 7.5), *p* = 0.10). Other univariate values are presented in Table 2.

**Table 2:** Detailed prognostic values for regional cerebral oximetry (rSO_2_), end-tidal CO_2_ (EtCO_2_) and pupillometry for survival at hospital admission.

**Parameter**	**Value for alive patients** ***(median [Q1; Q3])***	**Value for dead patients** ***(median [Q1; Q3])***	**Number of data included in the model** **(*n*, %)**	**AUC (95% CI)**	**Optimal threshold**	**Se (%; 95% CI)**	**Sp (%; 95% CI)**	**PPV (%; 95% CI)**	**NPV (%; 95% CI)**
**rSO_2_ (%)**									
Initial	30.0 (19.0; 43.0)	15.0 (15.0; 22.0)	89 (79.4)	0.74 (0.59–0.88)	29	62.5 (35.4; 84.8)	80.8 (69.9; 89.1)	41.7 (22.1; 63.4)	90.8 (81.0; 96.5)
Maximum	38.5 (28.3; 50.8)	15.0 (15.0; 30.3)	98 (87.5)	0.80 (0.69–0.92)	30	75.0 (47.6; 92.7)	72.0 (60.9; 81.3)	34.3 (19.1; 52.2)	93.7 (84.5; 98.2)
Median	29.5 (21.9; 42.5)	15.0 (15.0; 22.8)	98 (87.5)	0.79 (0.67–0.91)	23	75.0 (47.6; 92.7)	76.8 (66.2; 85.4)	38.7 (21.8; 57.8)	94.0 (85.4; 98.3)

**EtCO_2_ (mmHg)**									
Initial	43.5 (26.8; 56)	22.0 (15.0; 36.5)	86 (76.8)	0.77 (0.64–0.89)	25	92.3 (64.0; 99.8)	57.5 (45.4; 69.0)	27.9 (15.3; 43.7)	97.7 (87.7; 99.9)
Maximum	38.0 (32.0.0)	32.0 (17.5; 47.0)	94 (83.9)	0.71 (0.57–0.85)	26	100.0 (75.3; 100.0)	39.5 (28.8; 51.0)	21.0 (11.7; 33.2)	100.0 (89.1; 100.0)
Median	36.0 (25.0; 57.0)	25.5 (13.5; 36.0)	94 (83.9)	0.74 (0.60–0.87)	25	92.3 (64.0; 99.8)	50.6 (39.3; 61.9)	23.1 (12.5; 36.8)	97.6 (87.4; 99.9)

**Pupillometry (%)**									
Initial	6.0 (2.0; 11.5)	3.0 (0; 7.5)	100 (89.2)	0.65 (0.51–0.79)	5	61.1 (35.7; 82.7)	63.4 (52.0; 73.8)	26.8 (14.2; 42.9)	88.1 (77.1; 95.1)
Maximum	8.5 (2.3; 11.8)	8.0 (4.0; 13.5)	107 (95.5)	0.46 (0.30–0.61)	8	55.6 (30.8; 78.5)	49.4 (38.7; 60.2)	18.2 (9.1; 30.9)	84.6 (71.9; 93.1)
Median	4.0 (2.0; 4.0)	2.8 (1.4; 6.0)	107 (95.5)	0.55 (0.39–0.72)	10	33.3 (13.3; 59.0)	85.4 (76.3; 92.0)	31.6 (12.6; 56.6)	86.4 (77.4; 92.8)

*AUC: Area under the Curve; Se: Sensitivity; Sp: Specificity; PPV: Positive Predictive Value; NPV: Negative Predictive Value*.

### Analysis of initial rSO_2_, EtCO_2_ and pupillometry for the prediction of survival to hospital admission

**Fig. 2:**
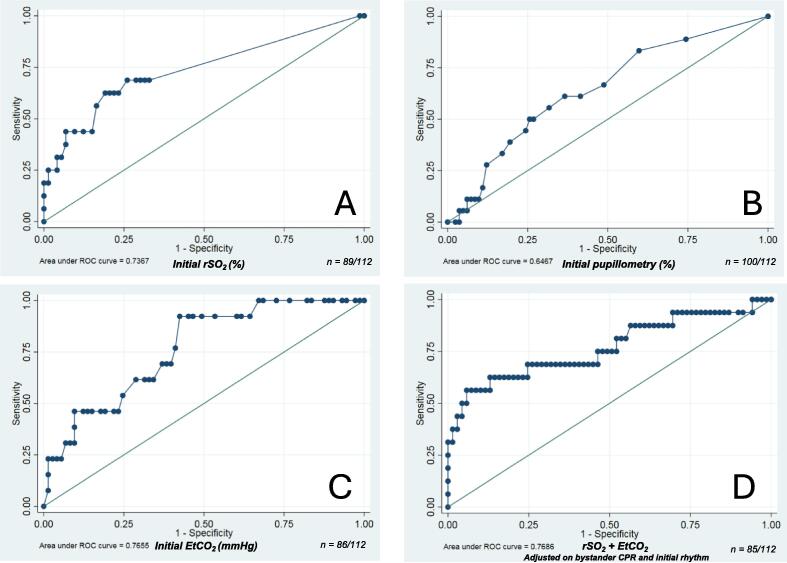
**Receiver Operating Characteristic (ROC) curve for initialnear-infraredspectroscopy (rSO_2_ (%)) alone (A), initial pupillometry (%) alone (B), initial end-tidal CO_2_ (EtCO_2_, mmHg) alone (C) and the association of rSO_2_ and pupillometry adjusted on bystander CPR and initial cardiac rhythm in a multivariate regression model (D)**. *n represents the number of data points included in the model. Detailed multivariate regression model is described in*Appendix 3*.*

For survival at hospital admission, initial rSO_2_ alone had an AUC of 0.74 (95% CI 0.59–0.88) with a sensitivity of 63%, a specificity of 81%, a NPV of 91% and a PPV of 42% (Table 2). The optimal threshold for initial rSO_2_ alone to predict hospital survival was 29%. All patients with rSO_2_ > 45% survived at hospital admission. Initial pupillometry alone had an AUC of 0.65 (95% CI 0.51–0.79) with a sensitivity of 61%, a specificity of 63%, a NPV of 88% and a PPV of 27% (Table 2). No optimal threshold was found for initial pupillometry. Initial EtCO_2_ alone had an AUC of 0.77 (95% CI 0.64–0.89) with a sensitivity of 92%, a specificity of 58%, a NPV of 98% and a PPV of 28% (Table 2). The optimal threshold for EtCO_2_ to predict survival at hospital admission was 25 mmHg. Patients with an initial EtCO_2_ ≥ 25 mmHg were 12 times more likely to be alive at hospital admission. The ROC curves are presented in Fig. 2. The predictive values of rSO_2_, EtCO_2_ and pupillometry in association are presented in Table 3.

**Table 3:** Predictive values for the combination of near-infrared spectroscopy (rSO_2_, %), end-tidal CO_2_ (EtCO_2_; mmHg) and pupillometry (%).

**Parameter**	**Number of data included in the model** **(*n*, %)**	**AUC (95% CI)**	**Se (%; 95% CI)**	**Sp (%; 95% CI)**	**PPV (%; 95% CI)**	**NPV (%; 95% CI)**
EtCO_2_ + rSO_2_	75 (67.0)	0.85 (0.73; 0.96)	25.0 (5.5; 57.2)	98.4 (91.5; 100.0)	75 (19.4; 99.4)	87.3 (77.3; 94.0)
rSO2 + pupillometry	87 (77.7)	0.75 (0.59; 0.90)	62.5 (35.4; 84.8)	84.5 (74.0; 92.0)	47.6 (25.7; 70.2)	90.9 (81.3; 96.6)
EtCO_2_ + pupillometry	86 (76.8)	0.81 (0.70; 0.92)	100.0 (75.3; 100.0)	53.4 (41.4; 65.2)	27.7 (15.6; 42.6)	100.0 (91.0; 100.0)
EtCO_2_ + rSO_2_ + pupillometry	75 (67.0)	0.86 (0.74; 0.98)	83.3 (51.6; 97.9)	79.4 (67.3; 88.5)	43.5 (23.2; 65.5)	96.2 (86.8; 99.5)
rSO_2_ + pupillometry adjusted on bystander CPR and initial shockable rhythm	85 (75.8)	0.77 (0.61; 0.92)	56.3 (29.9; 80.2)	94.2 (85.8; 98.4)	69.2 (38.6; 90.9)	90.3 (81.0; 96.0)

*AUC: Area under the Curve; Se: Sensibility; Sp: Specificity; PPV: Positive Predictive Value; NPV: Negative Predictive Value*.

Regarding primary analyses, studying the association of initial rSO_2_ and pupillometry adjusted on bystander CPR and initial cardiac rhythm, the AUC was 0.77 (95% CI 0.61–0.92) with a sensitivity of 56%, a specificity of 94%, a NPV of 90% and a PPV of 69% (Fig. 2). A multivariate logistic regression model was performed, including initial rSO_2_ and initial pupillometry (in mm and %) adjusted on bystander CPR (yes or no) and initial cardiac rhythm (asystole vs pulseless electrical activity). In the multivariate model, AUC was 0.77 (0.61–0.92) for survival at hospital admission. The details regarding the multivariate analysis are described in Appendix 3.

### Analysis of maximum and median value for each parameter

In addition, the maximum and median values were analyzed for each parameter, as described in Table 2. Regarding rSO_2_, the AUC was 0.80 (95% CI 0.69–0.92) for maximum rSO_2_ and 0.79 (95% CI 0.67–0.91) for median rSO_2_.

For EtCO_2_, the AUC was 0.71 (95% CI 0.57–0.85) for maximum EtCO_2_ and 0.74 (95% CI 0.60–0.87) for median EtCO_2_.

Finally, regarding pupillometry (%), the AUC was 0.46 (95% CI 0.30–0.61) for maximum pupillometry and 0.55 (95% CI 0.39–0.72) for median pupillometry.

### Time analysis of EtCO_2_ and rSO_2_

**Fig. 3:**
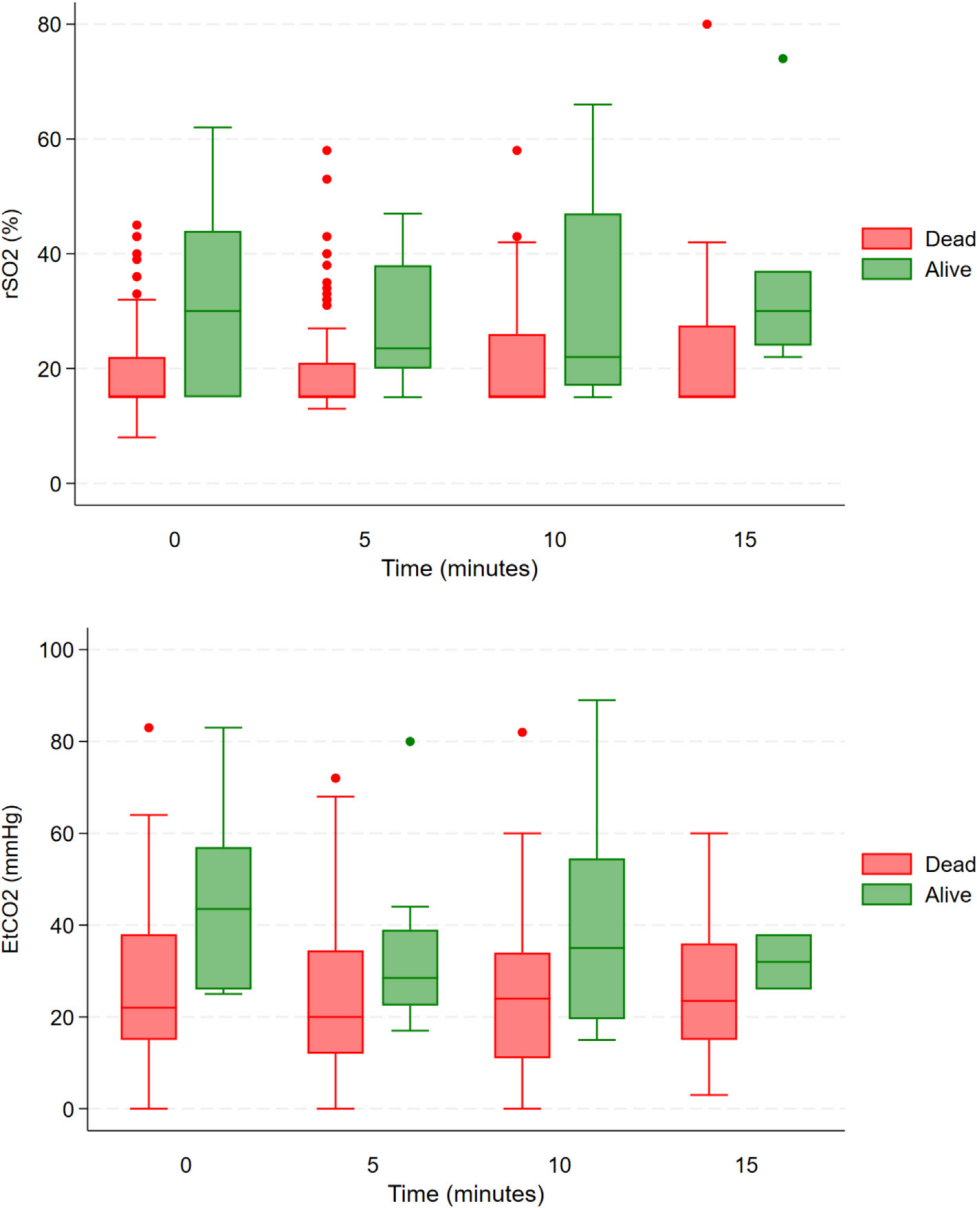
**Time analysis for near-infrared spectroscopy (rSO_2_) and end-tidal CO_2_ (EtCO_2_) for patients alive or dead at hospital admission**. *Means and standard deviation are presented for measurements at 0 min (T0), 5 min (T5), 10 min (T10), 15 min (T15), 20 min (T20)*.

A descriptive time analysis was conducted for EtCO_2,_ rSO_2_ and pupillometry measurements at prehospital team arrival (T0), five minutes (T5), ten minutes (T10), fifteen minutes (T15), and twenty minutes (T20) for patients who survived to hospital admission and those who were declared dead upon hospital arrival (Fig. 3 and Appendix 1). In the logistic regression model, there was no significant time trend effect in this population of rSO_2_ and EtCO_2_ on survival at hospital admission.

## Discussion

This prospective multicenter observational study demonstrated that the combination of EtCO_2_ and rSO_2_ may serve as important parameters related to survival at hospital admission in OHCA patients. Individually, both EtCO_2_ and rSO_2_ were also found to be relevant indicators of survival and mortality. Pupillometry demonstrated limited prognostic value in this small study population characterized by low survival rates.

Cerebral oximetry (rSO_2_), also known as near-infrared spectroscopy (NIRS), measures regional hemoglobin oxygen saturation in the brain using infrared light. It offers a novel, non-invasive, and user-friendly method for assessing cerebral oxygenation during cardiac arrest, requiring only surface application—unlike EtCO_2_, which necessitates advanced airway management for reliable measurements. The use of rSO_2_ in cardiac arrest is relatively recent, with feasibility studies emerging approximately a decade ago.^16^, ^17^, ^18^, ^19^, ^20^ A meta-analysis,^21^ suggested that an initial rSO_2_ below 30% is associated with mortality, proposed including this parameter in a multimodal approach for deciding when to terminate CPR. Conversely, higher rSO_2_ values have been observed in patients who achieve ROSC compared to those who do not.^22^, ^23^ Some studies also suggest that an increasing rSO_2_ trend during CPR may indicate ROSC.^23^, ^24^ However, rSO_2_ values between ROSC and no-ROSC groups often overlap significantly, with high specificities only at extreme values and generally low sensitivities,^25^ highlighted that rSO_2_ could play a role during CPR but with limitations, therefore not ready for routine clinical use. In the current study, rSO_2_ alone had an NPV of 91% and a specificity of 81%. Notably, all patients with a rSO_2_ > 45% survived to hospital admission. These findings suggest that while rSO_2_ has potential prognostic value, it is insufficient as a sole predictor. Therefore, this study combined rSO_2_ with EtCO_2_, resulting in an area under the curve (AUC) of 0.85 (95% CI, 0.73–0.96), achieving an excellent discrimination.^26^

EtCO_2_ remains the most established parameter for predicting survival in OHCA patients. According to the 2025 European Resuscitation Council (ERC) guidelines, an increase in EtCO_2_ during cardiopulmonary resuscitation (CPR) may indicate the return of spontaneous circulation (ROSC); however, chest compressions should not be interrupted based on this sign alone.^27^ A recent study found that an EtCO_2_ > 20 mmHg at the time of intubation, with a subsequent increase 10 min later, was predictive of survival to both hospital admission and discharge.^28^ Although EtCO_2_ shows promise as a prognostic factor, an optimal threshold has yet to be clearly established. Systematic reviews have recommended against using EtCO_2_ alone to predict survival in OHCA patients.^29^, ^30^ In the present study, EtCO_2_ demonstrated a high sensitivity (92.3%) and negative predictive value (NPV) (97.7%). The optimal threshold for predicting hospital admission survival was found to be 25 mmHg. Patients with an EtCO_2_ value >25 mmHg were 12 times more likely to survive to hospital admission. These findings are encouraging, but due to the low specificity and positive predictive value, additional parameters are needed for accurate survival prediction, as highlighted in the last ERC 2025 guidelines.^27^ Novel investigations on larger population, with assessment of multiple parameters and their potential association with outcomes alone and in combination, are needed.

In the present study, pupillometry was also assessed during the prehospital phase of CPR, prior to ROSC. The 2025 ERC guidelines recommend evaluating pupil size, symmetry, and light reactivity as part of post-cardiac arrest care, ideally 72 h or more after ROSC, to predict neurological outcomes.^31^ However, the guidelines do not address the use of automated pupillometry prior to ROSC.^27^ In this study, initial pupillometry yielded poor prognostic value for clinical prediction of survival to hospital admission, with an AUC of 0.65 (95% CI, 0.51–0.79), related to the poor pupil reactivity during ongoing chest compressions. Similar results were observed for both maximum and median values during CPR. These clinical findings contrast with experimental studies that have suggested a correlation between pupillometry, CPR quality, and prediction of ROSC.^32^ However, these results should be interpreted with caution. Most patients in the present study had non-reactive pupils at the onset of resuscitation, yet outcomes were poor, thereby limiting the impact of our findings. This highlights the need to further reassess the potential clinical role of pupillometry before ROSC.

### Limitations

This study has several limitations. First, the number of enrolled patients was limited due to the technical challenges of measuring three prognostic parameters under prehospital conditions. The mandatory time necessary to acquire these data (arrival of EMS team, endotracheal intubation, measure of infrared pupillometry and rSO_2_ placement and acquisition) excluded all the patient with the better prognosis (ROSC before ALS or in the early phase of ALS). Moreover, the exact time of assessment for each parameter was not recorded unless measurements were obtained immediately upon EMS arrival, thereby limiting the reproducibility of the results. The initial sample size target was 135 patients, based on an expected survival with favorable neurological outcome (Cerebral Performance Category [CPC] 1 or 2 at 30 days). During the study, the primary outcome was modified to survival to hospital admission, and the sample size was adjusted accordingly. These modifications were submitted to the institutional review board and registered on *clinicaltrials.gov*. While these changes may impact interpretation, they provide new insights into potential prognostic factors. Second, temporal changes in the three parameters (i.e., increasing or decreasing trends during CPR) were not statistically significant predictors of outcome. However, temporal analysis was not the primary objective and these results should be interpreted with caution. Third, blinded assessment of some parameters (pupillometry and rSO_2_) could have been occasionally difficult, and some measurements may have been unintentionally unblinded. Moreover, EtCO_2_ was not blinded to the entire team, including the physician, which could have influenced the decision to continue or discontinue CPR. Fourth, due to technical challenges in assessing parameters during CPR and a limited overall sample size, subject counts varied across the different models. Consequently, a direct statistical comparison between single-input and multimodal models (using DeLong’s test) was not performed. Future studies with larger, more consistent cohorts are needed to validate or refute these preliminary findings. Lastly, the study was conducted between 2016 and 2018 using older-generation devices. Newer technologies now available may offer improved measurement accuracy, potentially limiting the generalizability of these findings.

## Conclusion

During out-of-hospital cardiac arrest, near-infrared spectroscopy and EtCO_2_ values during chest compressions predict survival to hospital admission with acceptable discrimination. The combination of these two parameters enhances predictive accuracy, achieving a higher discrimination. Further studies are required to clarify the prognostic value of rSO_2_, EtCO_2_ and pupillometry for predicting survival at hospital admission.

## CRediT authorship contribution statement

**N. Segond:** Writing – original draft, Visualization, Validation, Formal analysis, Data curation. **D. Savary:** Writing – review & editing, Validation, Investigation, Data curation. **M. Veloso:** Writing – review & editing, Validation, Formal analysis, Data curation. **P. Bouzat:** Writing – review & editing. **J. Turk:** Validation, Investigation, Data curation. **J.L. Bosson:** Writing – review & editing, Validation, Methodology, Formal analysis. **D. Viglino:** Writing – review & editing. **J.F. Payen:** Writing – review & editing, Methodology. **G. Debaty:** Writing – review & editing, Validation, Supervision, Resources, Project administration, Methodology, Investigation, Funding acquisition, Formal analysis, Data curation, Conceptualization.

## Funding

The study was funded exclusively by Grenoble Alpes University Hospital.

## Declaration of competing interest

The authors declare that they have no known competing financial interests or personal relationships that could have appeared to influence the work reported in this paper.
