# Supplementary material for: Near-infrared spectroscopy, pupillometry and end-tidal CO2 as predictive factors of survival at hospital admission after out-of-hospital cardiac arrest
Source: Resusc Plus. 2026 Apr 9;29:101316. doi: 10.1016/j.resplu.2026.101316 (PMC13122785; doi:10.1016/j.resplu.2026.101316)
Supplement: Supplementary Data 1 [file mmc1.docx]

Near-infrared spectroscopy, pupillometry and end-tidal CO2 as predictive factors of survival for patients with out-of-hospital cardiac arrest.

**APPENDICES**

| **time (min)** | **0** | **5** | **10** | **15** | **20** |
| --- | --- | --- | --- | --- | --- |
| **rSO2 (alive)**  ***mean ± std (%)*** | 33.3 ± 15.3 | 27.3 ± 11.4 | 31.8 ± 19.8 | 37.4 ± 21.3 | 45.0 ± 18.5 |
| **No. rSO2 (alive)** | 15 | 10 | 10 | 5 | 3 |
| **rSO2 (dead)**  ***mean ± std (%)*** | 19.5 ± 8.4 | 20.0 ± 9.7 | 21.1 ± 9.3 | 23.1 ± 12.7 | 19.5 ± 8.0 |
| **No. rSO2 (dead)** | 72 | 71 | 65 | 53 | 20 |
| **EtCO2 (alive)**  ***mean ± std (mmHg)*** | 45.0 ± 20.1 | 34.6 ± 20.2 | 40.3 ± 26.5 | 32.0 ± 8.5 | 18.0 ± nan |
| **No. EtCO2 (alive)** | 10 | 8 | 8 | 2 | 1 |
| **EtCO2 (dead)**  ***mean ± std (mmHg)*** | 27.2 ± 17.1 | 25.7 ± 17.7 | 25.0 ± 16.2 | 26.3 ± 14.7 | 31.7 ± 16.7 |
| **No. EtCO2 (dead)** | 63 | 70 | 66 | 51 | 18 |
| **Pupillometry (alive)**  ***mean ± std (mmHg)*** | 11.9 ± 21.0 | 9.7 ± 15.8 | 8.7 ± 13.6 | 5.0 ± 5.8 | 7.3 ± 11.0 |
| **No. Pupillometry (alive)** | 15 | 11 | 12 | 5 | 3 |
| **Pupillometry (dead)**  ***mean ± std (mmHg)*** | 8.0 ± 18.7 | 8.4 ± 16.5 | 6.7 ± 13.0 | 5.6 ± 7.2 | 4.6 ± 5.4 |
| **No. Pupillometry (dead)** | 75 | 79 | 72 | 57 | 24 |

**Appendix 1** – Time evolution of rSO2, EtCO2 and pupillometry for patients alive or dead at hospital admission

*rSO2: near-infrared spectroscopy, EtCO2: end-tidal CO2; No.: number of available data.19 patients were alive at hospital admission and 93 were dead or died before hospital admission.*

|  | **All cases (n = 112)**  **Number of missing data; n (%)** |
| --- | --- |
| Age | 0 (0%) |
| Women | 0 (%) |
| Cardio arrest location | 1 (0.9%) |
| Witness cardiac arrest | 1 (0.9%) |
| Inclusion center | 0 (0%) |
| Type of witness | 3 (2.7%) |
| CPR initiated by witness | 2 (1.8%) |
| Etiology of cardiac arrest | 14 (12.5%) |
| Initial cardiac rythm | 2 (1.8%) |
| Signs of life (gasps) | 0 (0%) |
| Symmetric pupil at EMS arrival | 12 (10.7%) |
| Areactive pupil | 14 (12.5%) |
| Adrenaline dose | 0 (0%) |
| Amiodarone dose | 0 (0%) |
| Delay from cardiac arrest to firefighters’ arrival | 12 (10.7%) |
| Delay from cardiac arrest to first shock | 67 (59.8%) |
| Delay from cardiac arrest to EMS arrival | 7 (6.3%) |
| Delay from first call to firefighters’ arrival | 0 (0%) |
| Delay from first call to first shock | 0 (0%) |
| Delay from first call to EMS arrival | 0 (0%) |
| ROSC | 0 (0%) |
| Survival at hospital admission | 0 (0%) |
| Survival at hospital discharge | 0 (0%) |
| Survival at 30 days | 0 (0%) |
| CPC 1 or 2 at 30 days | 0 (0%) |

**Appendix 2** – Number of missing concerning population characteristics

*EMS: Emergency Medical Services; VF: Ventricular Fibrillation; VT: Ventricular Tachycardia; ROSC: Return to Spontaneous Circulation; CPC: Cerebral Performance Category status*

| **Parameter** | **AUC (95% IC)** | **Optimal threshold** | **Se (%)** | **Sp (%)** | **PPV (%)** | **NPV (%)** |
| --- | --- | --- | --- | --- | --- | --- |
| Initial rSO2 (%) | 0.74 (0.59-0.88) | 29 | 62.5% | 80.8% | 41.7% | 90.8% |
| Initial pupillometry (%) | 0.65 (0.51-0.79) | 5 | 61.1% | 63.4% | 26.8% | 88.1% |
| Initial pupillometry (mm) | 0.46 (0.29-0.64) | 7.1 | 11.8% | 95.2% | 33.3% | 84.0% |
| Bystander CPR | X | X | 84.2% | 38.5% | 22.2% | 92.1% |
| Initial Cardiac rhythm (asystole/PEA vs. fibrillation) | 0.61 (0.49-0.74) | X | 47.4% | 73.6% | 27.3% | 87.0% |
| Multivariate Model | 0.77 (0.61-0.92) | 0.39 | 56.3% | 94.2% | 69.2% | 90.3% |

**Appendix 3** – Multivariate model with the values included (initial value of rSO2, initial pupillometry (%), initial pupillometry (mm), bystander CPR, initial rhythm).

*AUC: Area under the Curve ; Se: Sensibility ; Sp: Specificity ; PPV: Positive Predictive Value ; NPV: Negative Predictive Value ; CPR: Cardiopulmponary Resuscitation ; PEA : Pulseless Electrical Activity*
